# Using a Smartwatch App to Understand Young Adult Substance Use: Mixed Methods Feasibility Study

**DOI:** 10.2196/50795

**Published:** 2024-06-20

**Authors:** Sahiti Kunchay, Ashley N Linden-Carmichael, Saeed Abdullah

**Affiliations:** 1 College of Information Sciences and Technology The Pennsylvania State University University Park, PA United States; 2 Edna Bennett Pierce Prevention Research Center The Pennsylvania State University University Park, PA United States

**Keywords:** smartwatches, substance use, ecological momentary assessment, mobile health, mHealth, human-centered design, feasibility studies, mobile phone

## Abstract

**Background:**

Young adults in the United States exhibit some of the highest rates of substance use compared to other age groups. Heavy and frequent substance use can be associated with a host of acute and chronic health and mental health concerns. Recent advances in ubiquitous technologies have prompted interest and innovation in using technology-based data collection instruments to understand substance use and associated harms. Existing methods for collecting granular, real-world data primarily rely on the use of smartphones to study and understand substance use in young adults. Wearable devices, such as smartwatches, show significant potential as platforms for data collection in this domain but remain underused.

**Objective:**

This study aims to describe the design and user evaluation of a smartwatch-based data collection app, which uses ecological momentary assessments to examine young adult substance use in daily life.

**Methods:**

This study used a 2-phase iterative design and acceptability evaluation process with young adults (aged 18-25 y) reporting recent alcohol or cannabis use. In phase 1, participants (8/15, 53%) used the data collection app for 14 days on their Apple Watches to report their substance use patterns, social contexts of substance use, and psychosocial risk factors (eg, affect). After this 14-day deployment, the participants completed a user experience survey and a semistructured interview to record their perspectives and experiences of using the app. Formative feedback from this phase informed feature modification and refinement of the app. In phase 2, an additional cohort (7/15, 47%) used the modified app for 14 days and provided feedback through surveys and interviews conducted after the app use period.

**Results:**

Analyses of overall app use patterns indicated high, consistent use of the app, with participants using the app for an average of 11.73 (SD 2.60) days out of 14 days of data collection. Participants reported 67 instances of substance use throughout the study, and our analysis indicates that participants were able to respond to ecological momentary assessment prompts in diverse temporal and situational contexts. Our findings from the user experience survey indicate that participants found the app usable and functional. Comparisons of app use metrics and user evaluation scores indicate that the iterative app design had a measurable and positive impact on users’ experience. Qualitative data from the participant interviews highlighted the value of recording substance use patterns, low disruption to daily life, minimal overall burden, preference of platforms (smartphones vs smartwatches), and perspectives relating to privacy and app use in social contexts.

**Conclusions:**

This study demonstrated the acceptability of using a smartwatch-based app to collect intensive, longitudinal substance use data among young adults. The findings document the utility of smartwatches as a novel platform to understand sensitive and often-stigmatized behaviors such as substance use with minimal burden.

## Introduction

### Background

Young adults exhibit some of the highest rates of substance use across all age groups in the United States [1], including alcohol use (50.2% or 17.5 million people), cannabis use (25.9% or 9 million people), vaping nicotine (24% or 8.3 million people), and prescription psychotherapeutic drug use (7.3% or 2.5 million people). Substance use can be associated with significant long-term effects on individuals’ health and well-being [2]. As such, there is an urgent need to understand, detect, and mitigate substance use among young adults.

There has been much prior work within the substance use domain in determining various psychological, social, and environmental factors that impact young adults’ substance use behaviors. These studies [3-6] highlight the value of collecting mood, affect, situational, and social context data to assess how they affect substance use patterns in this population. In recent years, this domain has shifted from relying on cross-sectional surveys and retrospective data toward using ecological momentary assessments (EMAs) on a daily level to detect relevant within-person trends. With recent advances in ubiquitous technologies and the surge of interest in accessible and affordable health care, there has been an increasing focus on understanding substance use and associated consequences through technology-based solutions. Thus, in the aforementioned studies [3-6], smartphones have been the primary device of choice.

In addition to having adaptable interfaces that support EMAs, smartphones also have extensive sensors that show potential to unobtrusively detect substance use in young adult populations. Prior work in the ubiquitous-computing community has described apps that seek to collect and analyze data to predict drinking episodes. Several studies have investigated the efficacy of inferring alcohol use through a smartphone user’s gait [7-9], as well as device use and movement features [10,11]. Smartphone sensors also exhibit potential in detecting cannabis use behaviors from users’ gait using accelerometer and gyroscope data [12], as well as from a combination of time features and GPS, accelerometer, SMS text messaging, and smartphone logs [13].

Systems that are capable of capturing behaviors, experiences, and sensor data in real time provide researchers a deeper understanding of the various contexts in which young adults engage in substance use. Although smartphones have been successful in collecting such data and are thus widely used in substance use research, a recent review of EMA protocols determined that compliance for substance use–related EMAs deployed on participants’ smartphones was lower than acceptable levels [14]. Hence, there is a need to explore novel interfaces and establish their utility in collecting granular substance use data with high compliance and low perceived burden.

Smartwatches offer a user experience that is distinct from that of a smartphone. The persistent, wearable nature of this device can enable users to observe cues (such as notifications, sounds, and vibrations) and perform quick interactions in diverse situations, such as when the smartphone is out of reach or an inconspicuous use of technology is required to minimize social disruption [15,16]. Moreover, smartwatches offer extensive health-sensing features that allow individuals to track and understand health behaviors. Thus, in recent years, there has been wide adoption of smartwatches: globally, approximately 202 million individuals own smartwatches [17], with 1 in 5 Americans using a smartwatch or fitness tracker [18]. This uptake of smartwatches by consumers has propelled researchers to investigate how smartwatches can be used as instruments of behavioral health studies. In fact, there have been several efforts to investigate whether illnesses and disorders could be recorded or managed through smartwatch-based tools such as those for managing attention-deficit/hyperactivity disorder [19] and posttraumatic stress disorder [20], aiding students with intellectual and developmental disabilities [21], assessing mobility among older adults [22], and managing chronic disorders [23]. In addition, there has also been interesting work in terms of detecting substance use behaviors, such as smoking, using these devices; for example, Skinner et al [24] used the accelerometer and gyroscope data in the Android Wear–based LG G Watch to detect signature hand movements of cigarette smoking.

Smartwatches and fitness trackers have met with resounding success in the health monitoring and self-management market [25,26]. Individuals use these devices to monitor and manage their fitness, sleep, mental health, and menstrual cycles through various apps. *Given their wide adoption for assessing health behaviors, especially by young adults [26], we argue that smartwatches may be well suited to understand substance use trends and patterns in this population*. In fact, Carreiro et al [27] highlight the significant potential yet underuse of wearables in combining detection and interventions for substance use. Importantly, the authors emphasized that wearable-smartphone combinations (such as smartwatches) are especially suitable for understanding and addressing substance use among adolescents and young adults. Recently, several studies pioneered the use of wearable sensors in understanding substance use and associated factors [28-30]. In these studies, participants noted several perceptions that suggested their preference for smartwatch-type interfaces over research-grade sensors for detecting and understanding substance use. Participants noted that these smartwatch interfaces were easy to integrate into their lives, offered various auxiliary features (such as screens, clock faces, and fitness-tracking capabilities), and drew minimal attention from strangers [28,29]. These aspects of smartwatches address many barriers that participants often face while using sensors and wearables in research studies. However, despite their potential and rapid uptake, these devices have rarely been used to assess substance use–related health behaviors in young adults.

### Objectives

There is a critical need to better understand and assess substance use behaviors and trends in real-world settings, and this need has so far been addressed by using smartphones for collecting self-report and sensor data. However, the engagement and compliance rates of smartphone apps in this domain are less than ideal, indicating a need to explore the suitability of other interfaces to collect such data. Therefore, this study aims to address this need by assessing the feasibility and acceptability of using smartwatches to collect EMA and sensor data to understand young adult substance use. Our use of smartwatches for this study is motivated by several reasons. First, in recent years, there has been wide adoption of commercially available smartwatches, specifically for health assessments and interventions. Second, smartwatches offer a novel user experience, built-in health sensor data capture, and popularity within young adult populations, thus offering the potential to collect richer, more granular data to understand young adults’ substance use with minimal burden. Finally, existing research in the substance use domain suggests that smartwatches may be especially suitable for understanding young adults’ substance use behaviors [27].

## Methods

### System Design and Development

Designing apps for smartwatches requires approaches and techniques that vary significantly from those required for typical smartphone app experiences. Smartwatch apps offer a seamless and intuitive experience when they are responsive; involve simple tasks; and make use of features that draw users to the device, such as haptic notifications, glanceable content, intuitive gestures, and a focused core functionality [31].

**Figure 1:**
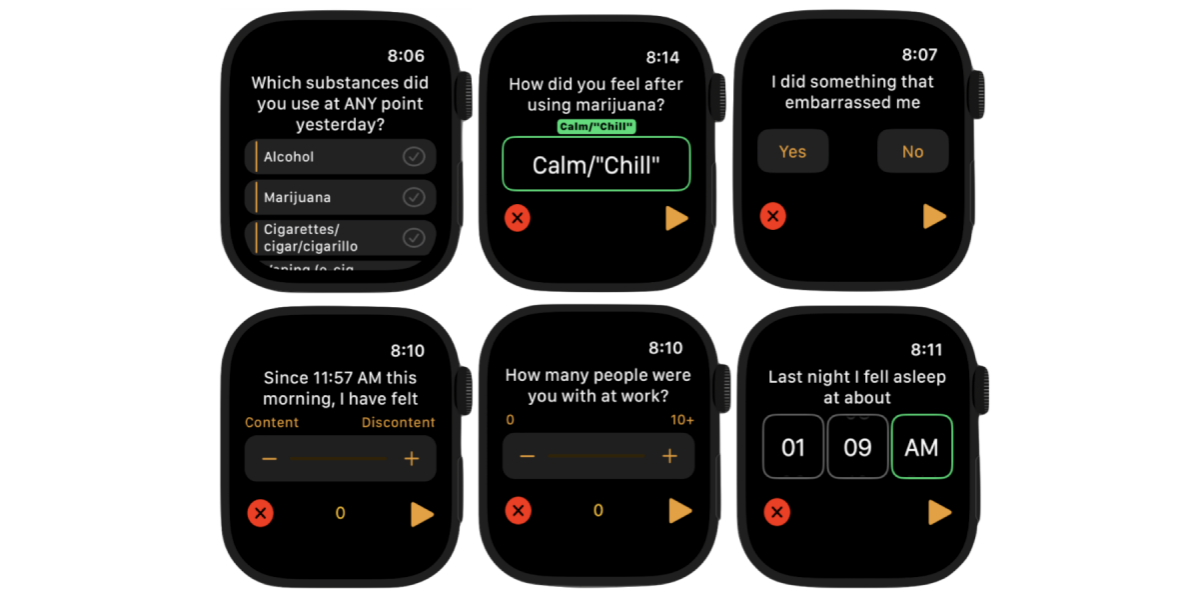
Screenshots of ecological momentary assessments on an Apple Watch interface depicting the variety of interface elements used to elicit responses from participants.

The primary requirement of the interface concerns ensuring that it provides an experience that results in highly granular and robust data collection, while keeping the perceived burden of interaction low. To address this challenge of high response rates and low study burden, we designed our questionnaire so that each question would take <5 seconds to answer. We expected that keeping the questions concise and interactions intuitive would help maintain low perceived burden and survey fatigue. Examples of these EMA components on the smartwatch (ie, an Apple Watch) are depicted in Figure 1. A companion app on the user’s iPhone uploaded all user responses and sensor data to our database.

### Iterative Application Design: Phase 1 and Phase 2

Our fundamental approach to app design and development was based on the principles of human-centered design, an approach that heavily incorporates users’ experiences and perspectives throughout the design process. It is a nonlinear process that iterates continually between various stages of understanding users, defining the problem domain, generating ideas, prototyping or developing solutions, and testing. This process helps build mobile health (mHealth) systems that are usable, effective, and accessible [32,33].

The creation of the smartwatch app went through continual iterations of user evaluation and development to produce an experience that enables robust data collection while also ensuring that the app is easy to use, minimally invasive, and considerate of users’ privacy and security concerns. Thus, we incorporated feedback from participants (8/15, 53%) in phase 1 of the feasibility study, so that participants (7/15, 47%) enrolled in the next phase were able to evaluate a refined app that provided a better user experience. During the initial rounds of testing and development, the app required confirmation from the database for each EMA question, causing a 1- to 2-second delay. This delay generated negative feedback from phase 1 participants. They reported that this delay between questions was frustrating and prompted them to assume that their responses were not recorded. To correct for this delay, we eliminated the step of waiting for the database confirmation before moving to the next screen.

### Participants and Procedures

Our methodology for the feasibility study was informed by two main objectives: (1) to fully capture participants’ experience using the smartwatch app and their perspectives on its usability; and (2) to collect data that accurately reflect users’ lived experiences with substance use, social contexts, affect, behaviors, and experiences. Hence, for this study, we used a mixed methods design with 3 key components: a 14-day in situ data collection period, where participants used the app to answer short EMAs regarding their behaviors and experiences; a poststudy survey that sought to quantitatively capture the usability of the app through various dimensions; and a semistructured interview that sought to capture more nuanced perspectives on participants’ experiences with the app.

To be eligible, participants needed to be aged 18 to 25 years, report past-week alcohol or cannabis use, own and use both an iPhone (with iOS version 15 or newer) and an Apple Watch (with watchOS version 8 or newer) to deploy and use the smartwatch app, and be a current student at the local university.

Participants were recruited through convenience sampling, using study flyers posted on the university campus, social media posts, and the university’s StudyFinder website. Potential participants were asked to email the study team if they were interested, after which they were sent a link to the screener survey as well as more details about the study. Informed consent to participate in the study was also obtained at this stage.

Eligible participants were immediately directed to a baseline survey in which they provided demographic information, typical substance use behavior, and technological use behaviors. The screener and baseline surveys were collected and managed using REDCap (Research Electronic Data Capture; Vanderbilt University) [34]. After completing the baseline survey, participants were scheduled for a web-based visit with the research staff who explained the research activities, guided them through app installation, and informed them about the compensation structure. Of the 25 eligible participants who completed the baseline survey, 15 (60%) scheduled and attended the web-based visit. After the completion of the 14-day data collection period, participants were requested via email to upload their HealthKit (Apple Inc) data, complete the usability survey, and schedule a second web-based visit for the semistructured interview.

Of the 15 participants who used the app, 12 (80%) completed all research activities. All study activities were conducted virtually between August 2021 and May 2022.

### Measures

#### EMA Data

The types of data we collected from the user through the EMAs related to (1) mood and general affect [35]; (2) experiences of stress; (3) sleep duration; (4) types and amounts of substances used; (5) feelings of intoxication [36,37]; (6) substance use–related consequences [38]; and (7) social context, such as location and social environment. The questions for self-reports explored a wide range of constructs and were sourced from prior research and findings that established their validity and reliability [35-38]. All constructs used in this app were motivated by a wealth of research indicating various associations with substance use [5,39-45]. A full list of all aforementioned EMA items is included in Table S1 in Multimedia Appendix 1 [35-38]).

Participants were sent 5 survey prompts per day at 11 AM, 4 PM, 7 PM, 10 PM, and 1 AM, which were available only for specific time windows or *sessions* every day (11 AM-3 PM, 4 PM-6 PM, 7 PM-9 PM, 10 PM-midnight, and 1 AM-3 AM, respectively). A brief overview of the initial design and development of this app is provided in prior work [46]. For every item, participants had the option of skipping the question if they did not wish to respond.

In the 11 AM session, participants were asked about their experiences and behaviors that occurred at any time on the previous day, and these data were grouped as *prior day* data while analyzing responses. Participants were also asked (in all sessions) about their experiences and behaviors that occurred since their last response, and these data were categorized as *periodic* data during analysis (Table S1 in Multimedia Appendix 1).

#### Sensor Data

In addition to self-report questionnaires, we also collected sensor data: location (GPS), physical activity, and health data streams. The health data streams serve various purposes: physical exercise, exercise intensity, and the types of exercise are all factors that have significant benefits in reducing substance use, decreasing depression symptoms associated with substance use, and improving the abstinence rate among those using illicit substances [44,45]; sleep has a bidirectional relationship with substance use in young adults, with sleep patterns and duration being significant predictors of cigarette, alcohol, and cannabis use; and the type of substance use is a significant predictor of total sleep duration as well as sleep patterns (eg, weekend oversleep) [43]. Although the limited sample size in this study hinders us from assessing whether these data streams can be effectively leveraged to unobtrusively detect substance use behaviors, the feature is incorporated into the app to examine preliminary associations, as well as for use in future studies with an anticipated larger sample size.

#### User Experience Evaluation

For the usability survey, we used the System Usability Scale (SUS) [47] to assess the perceived usability of the Apple Watch app, and we used an adapted version of the Mobile Application Rating Scale: User Version (uMARS) [48] and various other items to assess the acceptability of the interface and the EMAs sourced from prior work [49,50]. Both the SUS and uMARS surveys have high reliability and validity and have been extensively used to evaluate digital systems and mHealth systems, respectively.

In the semistructured interview, we queried the participants on whether the app impacted their substance use or substance cravings; whether the app influenced their awareness of substance use patterns; whether they had any concerns about using the app in various social contexts; and whether they had any privacy concerns regarding their substance use data, location data, or HealthKit data. All interviews were conducted via Zoom (Zoom Video Communications, Inc) and were recorded and transcribed using Zoom’s live transcription service powered by Otter.ai. The interview script is provided in Textbox S1 in Multimedia Appendix 1.

### Analysis

Only deidentified data were used during the analysis of app use data and interview data, blinding the authors to the identity of the participants while reviewing the results.

For our analysis, we focused on analyzing participants’ EMA responses, app use patterns, and user perspectives to determine the feasibility and acceptability of the smartwatch app. To understand the effect of the iterative design improvements, we compared various measures between participants from both phases, treating them as separate groups during analysis. These findings are discussed in the *Results* section.

### Ethical Considerations

All study activities and methods were approved by The Pennsylvania State University Institutional Review Board (17735) in the northeastern region of the United States in a state in which medical cannabis was legal, but recreational cannabis use was not legal at the time of data collection. A certificate of confidentiality was secured to protect participant responses concerning underage and illegal substance use behavior. All 15 participants provided informed consent before taking part in the study.

Participants were compensated for the study through Amazon gift cards and followed an established structure. Participants were compensated US $5 for completing the baseline survey, up to US $33 for the EMA data collection period, and US $10 for completing the user experience survey and semistructured interview. For the in-the-wild data collection period, participants were compensated US $2 per day if they completed both the 11 AM session and 1 other session during the day, but they were compensated only US $1 if they completed only the 11 AM session. Participants who did not complete the 11 AM session were not compensated for the day. If participants answered even 1 EMA during the 14-day period, they were compensated US $5.

## Results

### Quantity and Description of EMA Data Set

Participants ranged in age from 20 to 25 (mean 22.20, SD 1.86) years, and all were college students (undergraduate students: 10/15, 67% and graduate students: 5/15, 33%). Two-thirds (10/15, 67%) of the participants identified as female, while one-third (5/15, 33%) identified as male. Of the 15 participants, 5 (33%) identified as Asian, 1 (7%) as Black or African American, and 7 (47%) as White, while 1 (7%) participant preferred not to answer the question about race. Only 1 (7%) of the 15 participants identified as Hispanic or Latinx. Additional participant demographics are reported in Table S2 in Multimedia Appendix 1.

Overall, the 15 participants provided 4796 responses to EMA questions over 210 days. On average, the app collected 320 (SD 151; range 110-652) responses across all participants across all days of the study. Our data consisted of 45 prior-day (collected only at session 1) substance use reports, with a majority of reports mentioning alcohol use (alcohol: n=39, 87%; cannabis: n=12, 27%). We also collected 67 periodic substance use reports, which were reports collected in sessions 1, 2, 3, 4, or 5. Of these 67 periodic substance use reports, a majority included alcohol use, and a small portion included cannabis use, vape (e-cigarette or Juul e-cigarette) use, and cigarette use (reports of periodic alcohol use: n=49, 73%; reports of periodic cannabis use: n=13, 19%). Table 1 details all instances of substance use reported by the participants. Of the 15 participants, 3 (20%) did not report any substance use during the study.

**Table 1:** App use and substance use reports by participants.

Phase and participant			Total days participated (n=14), n (%)		Total sessions completed (n=70), n (%)		Days compliant (n=14), n (%)		Total EMAs^a^ answered, n		Longest consecutive use of app (days; n=14), n (%)		Prior-day substance use reports (n=45), n (%)		Periodic substance use reports (n=67), n (%)
**Phase 1**															
	P1	10 (71)		18 (26)		5 (36)		224		9 (64)		1 (2; alcohol)		1 (1; alcohol)	
	P2	8 (57)		27 (39)		7 (50)		271		8 (57)		2 (4; alcohol)		2 (3; alcohol)	
	P3	13 (93)		21 (30)		8 (57)		441		7 (50)		11 (24; alcohol: n=5, 45; cannabis: n=11, 100; vape: n=11, 100)		20 (30; alcohol: n=5, 25; cannabis: n=12, 60; vape: n=18, 90)	
	P4	13 (93)		25 (36)		6 (43)		259		10 (71)		1 (2; alcohol)		3 (4; alcohol)	
	P5	7 (50)		12 (17)		5 (36)		110		2 (14)		0 (0)		1 (1; other)	
	P6	14 (100)		55 (79)		14 (100)		559		14 (100)		1 (2; alcohol)		3 (4; alcohol)	
	P7	8 (57)		10 (14)		2 (14)		147		7 (50)		3 (7; alcohol)		4 (6; alcohol)	
	P8	14 (100)		36 (51)		12 (86)		424		14 (100)		4 (9; alcohol)		4 (6; alcohol)	
**Phase 2**															
	P9	14 (100)		36 (51)		10 (71)		371		14 (100)		0 (0)		0 (0)	
	P10	12 (86)		19 (27)		6 (43)		235		10 (71)		2 (4; alcohol: n=2, 100; cannabis: n=1, 50)		2 (3; alcohol: n=2, 100; cannabis: n=1, 50)	
	P11	14 (100)		28 (40)		13 (93)		380		14 (100)		9 (20; alcohol)		3 (4; alcohol: n=2, 67; cigarettes/cigar/cigarillo: n=1, 33)	
	P12	12 (86)		22 (31)		8 (57)		219		4 (29)		0 (0)		0 (0)	
	P13	14 (100)		26 (37)		10 (71)		289		14 (100)		0 (0)		0 (0)	
	P14	14 (100)		46 (66)		13 (93)		652		14 (100)		10 (22; alcohol)		22 (33; alcohol)	
	P15	9 (64)		21 (30)		4 (29)		215		9 (94)		1 (2; alcohol)		1 (1; alcohol)	

^a^EMA: ecological momentary assessment.

When analyzing the intensity of substance use, we found that participants reported an average consumption of 3.44 (SD 3.09; min=1, max=≥10) alcoholic drinks, with more positive (mean 3.82, SD 2.05; range 0-6) than negative consequences related to alcohol use (mean 0.33, SD 0.66; range 0-3), while participants reporting prior-day cannabis use reported consuming an average of 8.92 (SD 2.23; min=3, max=≥10) hits, with an average of 2.667 (1.370; range 1-6) positive consequences and no negative consequences related to cannabis use.

Periodic substance use reports also included measures that asked participants to describe how they felt after consuming alcohol or cannabis. For alcohol use, the options provided were *buzzed*, *tipsy/happy*, *drunk*, and *wasted*. Most reports of alcohol use described participants feeling *buzzed* (12/28, 43%), followed by feeling *tipsy/happy* (9/28, 32%) and feeling *drunk* (7/28, 25%). For cannabis use, the options provided were *calm/chill*, *relaxed*, *high*, and *stoned*. Most reports of cannabis use described participants feeling *calm/chill* (3/7, 43%) or *high* (2/13, 29%). Cannabis use reports also included the manner in which the substance was consumed. A majority of responses reported cannabis use through pipes (7/13, 54%) or vapes (5/13, 38%).

Participants were also asked about various aspects of their health daily. In session 1, participants were asked about prior-day stress levels and sleep duration. In all sessions, participants were asked about their mood since the last response.

Of the 149 self-reports received for session 1, a total of 148 (99.3%) self-reports contained responses related to stress. In 96 (64.9%) of these 148 self-reports, participants reported that stressful events did not occur. When asked to rate their prior-day stress levels on a scale ranging from 1 to 100, on average, participants reported a stress level of 33.920 (SD 22.181; range 0-90). With respect to sleep, the app collected 147 self-reports, where participants were asked when they went to sleep the prior day and when they woke up on the current day. On average, participants reported 7.290 (SD 1.859; range 0-11.167) hours of sleep. Finally, participants were asked to report their mood through 8 bipolar items, which garnered 3167 self-reports.

Overall, the data collected through the app consisted of a broad range of substance use behaviors and experiences, as well as a variety of health behaviors. This suggests that *participants are willing and able to share substance use data through smartwatches*, along with a variety of measures that have historically been associated with substance use in young adult populations.

### App Use

We first examined how regularly participants used the app to answer EMAs. Of the 15 participants, 6 (40%) responded to at least 1 prompt on all 14 days of the study. Most participants (11/15, 73%) responded on ≥10 days. On average, participants provided data on 11.73 (SD 2.60) days out of the 14 days of the study. For participants completing all activities of the study, the average number of days participated was even higher: 12.24 (SD 2.14). Table 1 lists EMA completion details across each participant.

We had 403 sessions with ≥1 EMA response. On average, participants provided data for 26.80 (SD 12.15) sessions. We instructed participants to complete the first session every day along with at least 1 other session. Using these criteria, the overall compliance rate was 59% (8.2/14). On average, participants were compliant for 8.20 (SD 3.67) days out of the 14 days of the data collection period.

Finally, we also examined consecutive app use—the longest consecutive *streak* of days where participants used the app to provide responses. The longest streak was 14 days: 6 (40%) of the 15 participants used the app every day during the study. The average streak across all participants was 10.00 (SD 3.96) days, indicating sustained engagement with the app for a majority of the study duration.

#### Contextual Variations in App Use

In this part of our analysis, we wanted to determine whether there were certain times and contexts in which participants were less likely to respond to prompts than others. Toward this effort, we explored how app use patterns varied with time, substance use, and social environments.

**Figure 2:**
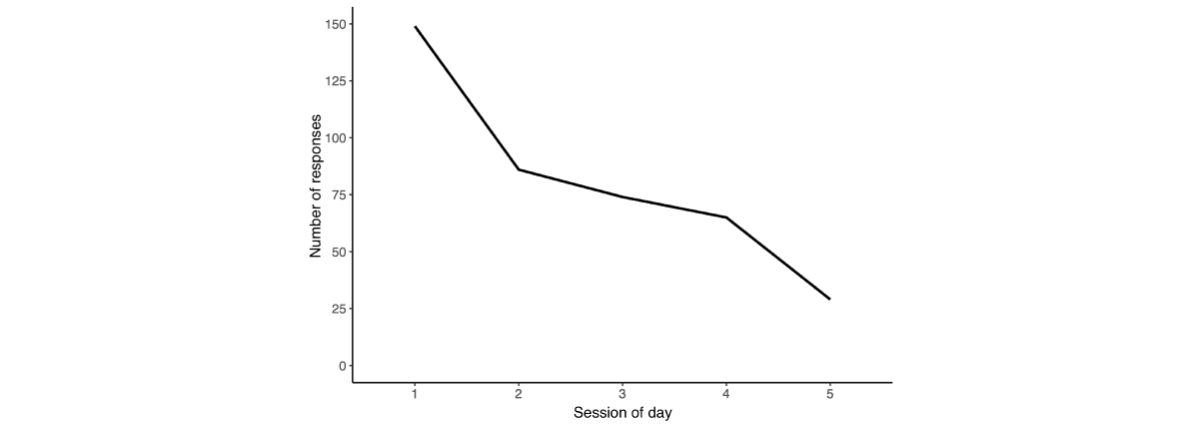
Variations in responses by session.

In our data set, the response rate varied across sessions (Figure 2). Session 1 (11 AM-3 PM) had the most responses, and app use fell as the day progressed, with the lowest responses being collected during session 5 (1 AM-3 AM). To understand whether the session of day had a significant effect on whether the participant would respond, we used multilevel modeling (using the *lme4* package in R).

A null model allowed us to calculate the intraclass correlation coefficient (ICC) of whether a participant responded. The ICC was 0.135, which meant that only 13.5% of the variation in responding stemmed from between-person differences, which indicated that a large proportion of the variation arose due to within-person changes. Thus, a random intercept model was created by adding the session of day as a predictor. This model significantly explains more of the variance in participants’ responses than the null model and hence is a better fit to the data (*χ*^2^_4_=181.6; *P*<.001). Using this model, we found that the session of day had a highly significant effect on whether the participant would respond. The odds and odds ratios calculated using this model indicated that the probability of a participant responding in session 1 was approximately 0.73. Compared to session 1, sessions 2, 3, 4, and 5 were respectively associated with a 76.47%, 82.29%, 85.88%, and 95.68% *decrease* in odds of a participant responding. In other words, *the probability of a participant answering in a particular session decreased significantly across the day*. Model details are described in Table S3 in Multimedia Appendix 1.

A random slope model did not significantly improve the fit of the model (*χ*^2^_14_=22.9; *P*=.06) and thus was not included for further analysis.

We used a similar method to understand whether the likelihood of reporting substance use varied across the day. The results of our null model calculated an ICC of 0.515, indicating that 51.5% of the variation in reporting substance use stemmed from between-person variances. The results from our model revealed that only session 5 had a significantly higher probability of participants reporting substance use compared to session 1 (estimate=1.70, SE 0.67; *P*=.01). The odds ratio for session 5 indicated that the odds of a participant reporting substance use in session 5 were approximately 5.49 times higher than the odds of a participant reporting substance use in session 1. This model proved to be a significantly better fit to the data than the null model (*χ*^2^_4_=11.3; *P*=.02), that is, participants were more likely to report substance use later in the day. Model details are described in Table S4 in Multimedia Appendix 1.

We also explored whether participants were able and likely to respond even when under the influence of substances. Specifically, we analyzed how participants’ responses differed after they reported substance use (compared with reports with no substance use). For this analysis, we used repeated measures correlations to determine within-individual association for paired or repeated measures data using the *rmcorr* package in R. We found no significant moment-level associations of substance use reports with responses in subsequent sessions, that is, whether a participant reported substance use in a specific session had no significant impact on their response to the first (*r*=0.02, 95% CI −0.08 to 0.13; *P*=.65), second (*r*=0.01, 95% CI −0.09 to 0.11; *P*=.83), third (*r*=0.03, 95% CI −0.07 to 0.13; *P*=.56), or fourth (*r*=−0.03, 95% CI −0.14 to 0.06; *P*=.46) session after the reported substance use. Random intercept multilevel models confirmed this result: reporting substance use in a specific session was not a significant predictor of whether a participant responded to the first, second, third, or fourth sessions after the session in question. Similarly, we saw no significant associations between social environments (people and places) and participants’ likelihood of responding.

To summarize, our findings suggested that *participants were likely to respond to EMA prompts in a variety of social contexts and after consuming substances*. However, we found a time effect, where participants were more likely to respond to prompts earlier in the day.

#### Differences in Use Patterns Between Design Phases

To investigate whether the improvements made to the smartwatch app had any effect, we compared 5 metrics between phase1 and phase 2 participants with respect to the total number of EMAs answered, the total number of sessions completed, the total number of days participated, the total number of days compliant, and the longest consecutive use of the app. Before running the analysis, we used the Shapiro-Wilk test to check whether the metric values were distributed normally across the phases. The distributions of counts from participants in phase 2 for the total number of days participated (W=0.77; *P*=.03), the total number of EMAs answered (W=0.74; *P*=.02), and the longest consecutive use of the app (W=0.77; *P*=.02) were all significantly nonnormal. Thus, to test for differences between the phases for these 3 variables, we used a nonparametric test, the Wilcoxon rank sum test. For the remaining variables, we used the independent 2-tailed *t* test (the Welch 2-sample *t* test) to examine whether the differences were significant.

Our analysis showed that, on average, the total number of days participated among phase 1 participants (mean 10.88, SD 2.95) was lower than that among phase 2 participants (mean 12.71, SD 1.89); however, this difference was not significant (W=17; *P*=.21; *r*=−0.32). Similarly, the group means were higher for phase 2 participants compared to those for phase 1 participants in terms of the total number of EMAs answered (phase 1: mean 304.38, SD 155.84; phase 2: mean 337.29, SD 154.78; W=21; *P*=.86; *r*=−0.05), the total number of sessions completed (phase 1: mean 25.50, SD 14.55; phase 2: mean 28.29, SD 9.64; t_12_=−0.44; *P*=.67), the total number of days compliant (phase 1: mean 7.38, SD 3.93; phase 2: mean 9.14, SD 3.39; t_13_=−0.94; *P*=.37), and the longest consecutive use of the app (phase 1: mean 8.88, SD 3.94; phase 2: mean 11.29, SD 3.86; W=17; *P*=.21; *r*=−0.33), but none of the differences between the phases were significant.

Although we did not see statistically significant increases in app use metrics after improving the app, the systematically higher engagement in terms of days used, EMAs answered, days compliant, and longest consecutive use suggests that the changes were a step in the right direction.

### User Evaluation

For our analysis of the user experience survey deployed after the participants finished their 14-day data collection period, we primarily focused on reporting various measures of usability, describing notable user perceptions, and comparing usability metrics between phase 1 and phase 2 participants to evaluate the effect of app improvements on overall user experience.

#### SUS Scores

Of the 15 participants who used the app, 12 (80%) completed all research activities. The average SUS score for all 12 participants was 63.54 (SD 18.78). As a comparison point, Bangor et al [51] found that the mean SUS score from 964 usability tests across various interface types was 70. However, a usability study of fitness trackers found that the average SUS score for an Apple Watch interface was 61.36 [52]. While slightly higher than average in terms of smartwatch interface, this score does provide the opportunity to understand pain points within the app. The mean score for each SUS measure is depicted in Table 2.

**Table 2:** Summary of itemized System Usability Scale (SUS) scores presented overall (combining the results of participants from both phases) and by study phase. Notably, phase 2 participants reported higher mean SUS scores than phase 1 participants, but this difference was not significant.

Measures	SUS items, mean (SD)											Total SUS score, mean (SD)
	1	2	3	4	5	6	7	8	9	10		
Overall	2.83 (1.11)	2.5 (1.17)	3 (1.28)	1.83 (0.94)	3.33 (1.30)	2.67 (1.44)	4.33 (0.49)	3 (1.35)	3.67 (1.07)	1.75 (0.96)	63.54 (18.78)	
Phase 1	2.17 (1.17)	3 (1.26)	2.83 (0.98)	1.5 (0.84)	3.33 (1.63)	3.33 (1.63)	4.17 (0.40)	3.33 (1.63)	3.33 (1.21)	1.83 (1.16)	57.08 (22.77)	
Phase 2	3.5 (0.55)	2 (0.89)	3.17 (1.60)	2.17 (0.98)	3.33 (1.03)	2 (0.89)	4.5 (0.55)	2.67 (1.03)	4 (0.89)	1.67 (0.82)	70 (12.55)	

**Figure 3:**
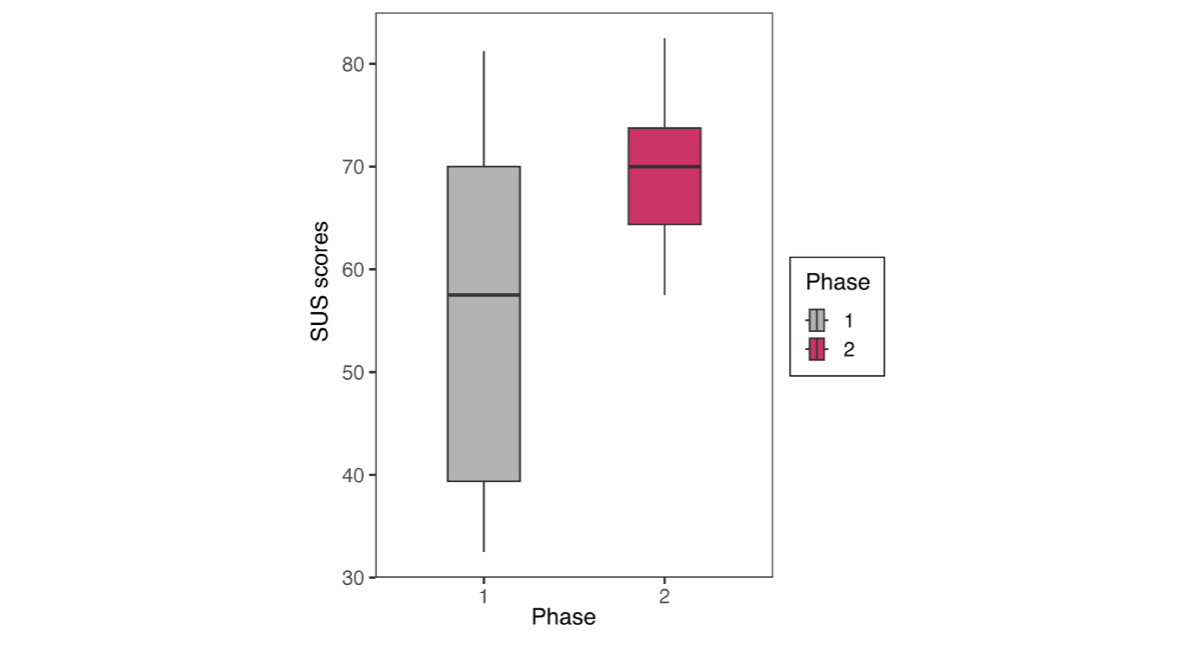
Box plot of System Usability Scale (SUS) scores by phase of study. Mean SUS scores were higher in phase 2, after we had made changes to the app to correct for delay issues. However, the difference in SUS scores between the phases was not statistically significant.

Before comparing overall mean SUS scores between the phases, we first used the Shapiro-Wilk test to check whether the SUS scores were distributed normally across the phases. The results of this test and an examination of skew and kurtosis values indicated that the SUS scores were distributed normally overall and by phase. Thus, to compare the means, we used the independent *t* test (the Welch 2-sample *t* test). *Although participants in phase 2 reported higher mean SUS scores than those in phase 1, this difference was not statistically significant* (phase 1: mean 57.08, SD 22.77; phase 2: mean 70.0, SD 12.55; *t*_8_=−1.21; *P*=.26). A box plot depicting differences in the SUS scores between the phases is shown in Figure 3.

#### uMARS Scores

All uMARS items were rated on a scale ranging from 1 to 5. Both the average functionality and aesthetics scores for all 12 participants were 3.88 (SD 0.55) and 3.89 (SD 0.52) respectively, indicating that participants found both measures acceptable (mean ≥3).

In terms of overall mean scores, participants in phase 2 rated app functionality as *good* (mean ≥4), while those in phase 1 rated app functionality as *acceptable*. Participants from both phases rated aesthetics as *acceptable*. Mean functionality scores were higher in phase 2 (phase 1: mean functionality score=3.58, SD 0.61; phase 2: mean functionality score=4.17, SD 0.31; *t*_7_=−2.11; *P*=.07), but mean aesthetics scores were slightly higher in phase 1 (phase 1: mean aesthetics score=3.94, SD 0.71; phase 2: mean aesthetics score=3.83, SD 0.28; *t*_7_=−2.11; *P*=.07).

Notably, the scores for the performance domain of the functionality metric from phase 2 participants exceeded those from phase 1 participants (phase 1: mean performance score=2.33, SD 0.52; phase 2: mean performance score=4.17, SD 0.75; difference in scores between the 2 phases=1.84). Using the Wilcoxon rank sum test, *we found that phase 2 participants rated the app significantly higher on the performance scale than phase 1 participants* (W=1; *P*=.006; *r*=−0.79). There were no significant differences between the phases for the ease of use, navigation, and gestural design domains of uMARS functionality scores. Similarly, none of the aesthetics domains (layout, graphics, and visual appeal) had any significant differences in ratings between the phases.

#### EMA-Specific Participant Perceptions

Along with ratings of established usability scales, we also asked participants specific survey questions about EMA usability, touching upon the constructs of the ease of use, enjoyment, the speed of answering EMAs, EMA length, interruptibility, and notions of trust and privacy.

Most of the participants (8/12, 67%) either agreed or strongly agreed that the EMAs were easy to fill. However, there were discrepancies between the phases. Most participants in phase 2 (5/6, 83%) agreed or strongly agreed that the EMAs were easy to fill, while 3 (50%) of the 6 participants in phase 1 either disagreed or strongly disagreed with this statement. Similarly, all participants in phase 2 (6/6, 100%) agreed or strongly agreed that they were able to complete the EMAs quickly, while 4 (67%) of the 6 participants in phase 1 disagreed with this statement. Of the 12 participants, 8 (67%) did not think that the EMAs were too long, and among the 4 participants who did think so, a majority (n=3, 75%) were phase 1 participants.

To examine perceived burden and fatigue, participants were asked about the number of days after which they felt tired of answering the EMAs. On average, participants in phase 1 reported fatigue after 6.40 (SD 3.44; range 3-12) days, while participants in phase 2 reported fatigue after 9.17 (SD 3.13; range 4-12) days. This difference among the phases was not statistically significant (*t*_8_=−1.39; *P*=.20).

Overall, most of the participants felt that the app was acceptable and simple to use. A higher proportion of participants in phase 2 felt so compared to those from phase 1. None of the participant ratings of ease, speed, or fatigue were significantly different across the phases, but the higher ratings in phase 2 suggest that the app changes were a step in the right direction to improve app usability and address critical issues.

### Interview Themes

#### Overview

In this subsection, we present our main findings from our analysis of the semistructured interviews that were conducted after the 14-day data collection period. For the analysis of the interview data, we used inductive thematic analysis to identify common themes using a qualitative interpretivist approach. The primary author conducted the initial analysis and then discussed the themes and codes with the other authors to ensure the validity of the primary findings and to reduce bias.

Overall, participants agreed that their experience using the smartwatch app to answer EMAs was easy, novel, and acceptable, but they also brought up certain key issues with app responsiveness and commented on the suitability of the smartwatch interface for this specific use case.

#### General App Perceptions

When asked about their overall experience using the app to answer EMAs, most of the participants shared that the app was generally easy to use. A participant recalled that using the app was quickly incorporated into their day, while noting that this was not disruptive to their routine:

I mean it kind of turned into, like, an everyday routine where, like I just expected it at certain times and I used to take time out and do it.

P6

This sentiment was echoed by another participant:

Since it is only like 3 to 5 minutes, I didn’t think that’s a very disruptive time point, like I could do it in between class or, if I was at dinner [or] lunch and I remembered, I’d typically do it then.

P4

Although not disruptive to their daily lives, this participant shared that using the app was different to how they normally used their smartwatch:

Disruptive? Not really. I don’t normally look at my watch for more than a couple of seconds, so that was a little different, but overall it wasn’t really that disruptive.

P4

Other participants also noted how completing the EMAs only took a few minutes (generally <2-3 min), unless lagging or responsiveness issues occurred.

#### Advantages and Challenges of Using Smartwatch Interfaces

Participants presented varied perspectives when it came to the elements of the smartwatch interface; for instance, some of the participants found advantages and preferred the fact that the smartwatch provided a small screen and a personal experience:

I think that’s the one benefit that the watch did have, is that it’s such a small screen that it’s hard for anyone to, you know, look at what you’re doing, on such a small screen. So the watch definitely had a benefit in kind of, like, protecting your privacy.

P8

By contrast, participants also noted that the small screen and the wearable experience presented a hindrance, with a participant sharing the challenges they faced in using the watch to answer EMAs:

Well, so for me, just having, just turning my arm and touching my watch is, I don’t know if it’s a range of motion thing, It’s just not the most natural thing to me and so just having to be in this position, looking at my watch, touching stuff, I don’t particularly like that.

P3

This participant indicated that a bigger screen would provide a more comfortable experience:

Just having a larger screen to be able to do everything on, I think it’d be a lot easier.

P3

Personal preferences factored greatly into how easy and intuitive participants found various aspects of the app experience. When asked about their perspectives on the various formats in which the questions were presented, such as sliders, checkboxes, and radio buttons, the responses were similarly varied. Some of the participants found all question formats easy to answer:

I think all of the formats were very straightforward and in terms of them, like, how they worked, I think they all worked just fine. There was no issue transitioning between the different formats.

P8

Others reported issues with the radio button and checkbox formats:

I think the multiple select got harder because just, like, being able to see all the options and then be able to click next on an Apple Watch screen [that] is kind of tiny, so in that sense, yes [was a difficult format to answer].

P7

Similarly, a participant faced challenges with the slider format:

Think the [slider] one, because I think I had to press, if I’m like, you know, perfectly energetic [on the MDMQ] then I had to go all the way plus plus plus plus plus, it was like, a lot of plusses. Other than that, the rest was great.

P12

#### App Responsiveness and Lagging Issues

Phase 1 participants frequently shared their experiences with recurring lag issues, noting that it lengthened app use time and caused disruptions and general frustration:

Overall, it was pretty easy and straightforward, but it did start to get frustrating switching between different survey prompts. It would get, like, frozen a lot. So I would click to go to the next prompt, I guess, and it would get frozen, so surveys that were supposed to take 2 minutes ended up taking upwards of 10 minutes because it would get frozen.

P8

A lot of the buttons weren’t the most responsive, so you had to click them a couple of times before it would actually do anything. And sometimes I had to restart my watch because it just wouldn’t have responded.

P3

As mentioned in the *Iterative Application Design: Phase 1 and Phase 2* subsubsection in the *Methods* section, we identified that this delay was caused by the data-uploading mechanism, which was corrected for phase 2. As a result, phase 2 participants did not report this frequent lag between questions in their interviews.

#### Comparisons to iPhone Platform

Several participants believed that having the option to answer the EMAs on both the iPhone and the Apple Watch would offer an easier and more seamless experience and provided the strengths of both devices to support this sentiment. A participant offered some context where such a system would prove useful for them:

So I know in the evening, sometimes, especially when I’m just, like, sitting on my couch, laying down, watching TV, I’ll take my watch off to charge for the night, but I’ll still have my phone with me. So, I’m not gonna get those alerts, if I’m not wearing my watch. So, it’s nice to be able to switch, then, to the different interface on my phone, to use that.

P14

Another participant shared a similar perspective:

I guess, that [having the option to complete surveys on both devices] would be okay, because that way you can at least see the surveys, do on your watch, in case your phone is not in your hand, you still have the watch, you’re wearing your watch, you have the option of both.

P11

By contrast, a participant shared a scenario where using a smartwatch would prove easier than using a smartphone:

Usually you have to open up the phone and then you have to take off your mask [to unlock it using facial recognition]. With the watch, you don’t have to do anything, you just, you know, do with the 1 finger, which makes it a lot easier and better.

P12

Similarly, another participant noted as follows:

I check my watch more than I check my phone. I feel just time wise, and yeah, I feel like it’d be harder to use the phone. Like take my phone out and use it.

P14

However, most of the participants agreed that the larger screen size would provide a smoother experience while answering EMAs, with a participant sharing their perspective on how having a bigger screen would benefit their experience:

Just cause it’s a little bigger, and you can just, like, do it on your phone while walking or something, and like on your watch you can’t really do that.

P10

#### Self-Monitoring Substance Use

Several participants shared how using the app provided a valuable self-tracking experience that helped them think about their substance use patterns. Although this was not an intended use of the app, participants found a tangible benefit in keeping track of their substance use to answer the EMAs accurately:

It makes you cognizant of your usage, and it makes you cognizant, while looking at the questions, as to what, you know, could be impacted [by substance use].

P11

A participant shared how answering the EMAs helped them evaluate their substance use:

I think it just forced me to kind of analyze...like, I’d mainly only drink on the weekends, so it made me [think about] how I spend my weekends and how much I was using a substance in a specific time frame. So it made you kind of take a step back and analyze that, which is always, I think, shocking to people, how much or how little they may have been using a substance.

P8

Answering frequent EMAs about their substance use helped participants increase their awareness of their substance use patterns and behaviors.

A participant also shared an interesting perspective of how useful they found the self-monitoring aspect of using the app and how they experienced a lack of incentive to track their substance use after the 14-day data collection period ended:

I think, just being aware of, like, how many drinks I was consuming. Yeah, because if I don’t have to track it, I don’t remember how much I drink. So, because I was able to be like oh, like the next window is at 7 o’clock, like, my next notification at 7, like that. I’ve had to remember that I’ve had, you know, 2 drinks to put it in that notification.

P14

Other participants noted how they already mentally keep track of their substance use, but using the app made them reevaluate their use:

It definitely increased my awareness, but I felt like I already knew. If I had work or most of the school days, like, I won’t be doing anything like that [substance use], but, more on the weekends. Like, oh, maybe I shouldn’t do this tonight, or something like that.

P7

Participants used the EMAs to reflectively track their substance use. These interactions augmented their existing self-monitoring practices to periodically and contextually evaluate their substance use behaviors.

#### Use of the App in Social Settings

All participants reported that they used the app in public and social settings and were comfortable doing so; for instance, a participant shared how their friends felt when they saw the participant using the app:

Yeah, like I thought it was totally fine. All my friends knew I was taking, [and] like I didn’t care that they knew. But when I was out at the bars, I was fine taking the surveys, and I don’t know if other people knew that I was using my watch, or whatever. But all my friends knew, and they thought it was cool.

P14

Another participant also spoke about their use of the app in such settings:

Yeah, like, if I got the notification when I was at school, like in class or something, or like walking to class, I would take it then.

P7

This indicates that the app is able to effectively collect data in various social settings. This finding is especially meaningful, given the sensitive and often-stigmatized nature of the substance use data that the app collects. The convenience and comfort with which participants are able to share information indicates that using the smartwatch in this way is potentially unintrusive in various social contexts and environments.

Several participants offered insight into how they did not have concerns regarding privacy or security while interacting with the app and shared how the smartwatch platform helped in this aspect:

No [I did not feel uncomfortable using the app in public or social settings]. I mean, the watch screen is so small, I don’t even think anyone realized what I was doing, that I’m on it.

P7

The small screen of the watch ensured that the participants’ activities while using the app remained private from their peers and other people in their vicinity and thus helped their perception of the security of their data.

## Discussion

### App Feasibility and Acceptability

Overall, the app collected 4796 responses to EMA questions from 15 participants over the course of a 2-week-long study. Participants demonstrated high and consistent use of the app, responding on an average of 11.73 (SD 2.60) days and consistently using the app for an average of 10 (SD 3.96) days. Our analysis of app use patterns indicates that participants respond in a variety of contexts: after they consume substances and among different social contexts. The interview data supported these findings: participants were able to quickly incorporate using the app into their daily life and easily provide substance use data, and they were comfortable using the app in diverse social settings. Together, these findings demonstrate that it is indeed feasible to use a smartwatch app to collect substance use data.

With respect to app use, the decrease in participants’ responses across the day was an interesting finding. We speculate that the higher response rate in session 1 might be due to the longer availability compared to other sessions (4 h vs 2 h). Participants were also specifically asked to complete session 1 each day and were compensated accordingly. However, our findings also indicate that participants were more likely to report substance use at night, in session 5 (1 AM-3 AM), than in session 1 (11 AM-3 PM), which coincides with substance use patterns among young adults. Together, these results suggest that there are certain time periods that may be better suited to obtaining specific insights into substance use behaviors. Morning and noon may be suitable periods to understand prior-day substance use behaviors, mood, and experiences, while late night might be better suited to understand evening drinking behaviors. As such, there is an opportunity to develop better informed and less burdensome methods for collecting substance use data*.* Future work should try to replicate our findings regarding the temporal variation of EMA completion rates for substance use.

In terms of user evaluations, the average SUS score for the 12 participants who completed the survey was 63.54 (SD 18.78). Participants in phase 2 reported higher mean SUS scores than those in phase 1 (phase 1: mean 57.08, phase 2: mean 70.00). For context, an SUS score of 70.00 is considered average and acceptable, but it is to be noted that this subjective qualification of SUS scores does not consider smartwatch interfaces. If we factor the interface into our assessment of participants’ SUS scores, we can estimate that overall and in phase 2, participants rated the app above average in usability. Furthermore, in terms of mean uMARS scores, participants from phase 2 rated app functionality as good (mean ≥4), while those from phase 1 rated app functionality as acceptable. Although not significant, these findings suggest that the performance improvements to the app had a large and measurable impact on participants’ perceptions of usability. Indeed, the improvement also had an impact on app use: on average, the total number of days participated, the total number of EMAs answered, the total number of sessions completed, the total number of days compliant, and the longest consecutive use of the app were all higher among phase 2 participants than among phase 1 participants.

These findings not only establish the user acceptability of smartwatches to collect substance use data but also indicate that app performance, specifically responsiveness to user inputs, is critical for user acceptance. Given the limited computational capability of smartwatches, it is particularly important to aim for responsive design by default. Modifications to improve app responsivity resulted in better perceived usability and user satisfaction, along with systematically higher user evaluation scores and app use metrics. Thus, supporting quick, responsive interactions is a critical consideration when designing EMAs for smartwatches. Researchers and practitioners interested in using these devices as platforms for intensive data collection must focus on efficient, quick, and simple interactions to ensure sustained use as well as acceptable compliance and response rates.

### Smartwatches and Substance Use

Our data consisted of 45 prior-day and 67 periodic substance use reports which contain alcohol, cannabis, cigarette/cigar/cigarillo, and e-cigarette/vape use data. Participants were able to share data on a range of variables associated with substance use through the smartwatch.

Furthermore, our interview data highlighted a key benefit that participants found through regularly using the app: tracking and reflecting on their substance use. Using the app to provide substance use data encouraged participants to contemplate on their substance use by requiring them to recollect aspects of their use (when, how much, with whom, etc). Even without a feature that displays the patterns of use, participants noted how the task of recollecting and entering substance use data helped to make them more aware of patterns within their substance use as well as cognizant of the contexts in which they consume various substances. While the benefits of self-monitoring substance use are not limited to the smartwatch interface, it is promising that a smartwatch app is able to successfully promote such experiences.

An aspect of the smartwatch interface that might have helped participants share substance use data confidently is the privacy that it affords through a smaller, more discreet screen. Participants reported how they felt comfortable using the app in social and public settings, saying that the small screen ensured that others in their vicinity would not be able to discern what the participants were doing on their smartwatch. Nevertheless, some participants thought that a larger screen, such as a smartphone screen, might be useful in certain contexts. Participants also noted that some question formats, such as those that require scrolling, are harder to complete on a small screen. Importantly, participants preferred having the option to complete a survey on a smartphone or a smartwatch, depending on what is most convenient at a given time and place. Future studies using smartwatches for health assessments and interventions should ensure that the proposed systems can work comfortably across diverse contexts. One way to accomplish this is by supporting interchangeable use of the app on different devices: smartphones *as well as* smartwatches. Users can then choose which device is most appropriate for their current activity and social environment and use the app correspondingly.

On the whole, our analysis of app use, surveys, and interview data indicate the feasibility and acceptability of using smartwatches in this domain, demonstrating that users are able and willing to use a smartwatch to share substance use data. Participants shared data on a range of substances, experiences, and behaviors and identified aspects of the smartwatch interface that enabled them to do so comfortably. Participants found that comprehensively self-monitoring their substance use through the app was a useful and important feature. Our findings also provide insight into which aspects of the smartwatch interface elicit responses as well as those that do not: while the small screen affords users privacy while relaying sensitive information such as substance use data, it can provide challenges for certain EMA formats and in certain contexts.

### Limitations

This study has a number of limitations that are important to discuss, given their potential impact on our findings. First, the study had a small sample size, which we considered to be acceptable, given that the goal of the study was to establish the feasibility and acceptability of a smartwatch-based app for collecting substance use data. However, we acknowledge that the reported findings may not be generalizable to the larger population of young adults who consume substances. Reproducing this study with a larger, more diverse sample can offer a wider perspective on the use of smartwatch-based apps to collect longitudinal, intensive data in this domain. The findings concerning significance should be interpreted with caution, given our small sample size and unstable estimates. Furthermore, participants were already smartwatch owners, which might have had an impact on the perceptions of usability. Thus, understanding the perspectives of novice smartwatch users using the app can help us investigate the effect of novelty on user experience. Finally, most participants in our sample were not binge drinkers or did not exhibit high-intensity substance use. The user experience of the app might differ with participants and circumstances that arise from heavy or hazardous substance use behaviors that are not adequately represented in our sample. Future work focusing on users who exhibit such patterns of substance use can help build more robust systems that cater to a wider range of people who use substances.

Next, we detail limitations associated with our app. Developing data collection apps on the Apple system has the constraint of being platform dependent (limiting the devices on which the apps can be deployed); however, developing for a single ecosystem was the first step in testing the general feasibility of a smartwatch-based data collection app. Implementing the data collection app on multiple ecosystems and running studies with various devices and apps was outside the scope of this study. However, our design and development process focused considerably on creating a reproducible and well-documented codebase so that cross-platform or platform-agnostic implementation can be achieved at a later stage.

### Conclusions and Future Work

In recent years, there has been wide adoption of smartwatches for health assessments and interventions. This paper focuses on ascertaining the feasibility and acceptability of using a smartwatch app to collect substance use data from young adults. Our data indicate that it is feasible and acceptable to use smartwatches to collect data about sensitive and stigmatized behaviors, including substance use. On the basis of these findings, we also discuss considerations for future smartwatch apps for health and well-being data collection. These findings have important implications for researchers aiming to leverage smartwatches as an mHealth platform for effective assessments and interventions. In the future, we plan to conduct a larger study, with a randomized between-participants experiment design, to compare app use and user perceptions between smartphones and smartwatches. This future study will help us understand which device results in better compliance, better engagement, and lower perceived burden within the context of substance use data collection. We also intend to use the health sensor data from this larger study to explore whether they can be used to unobtrusively detect substance use or associated behaviors. Finally, we aim to incorporate analyses such as the impact of battery life on app use to gain a nuanced understanding of how smartwatch capabilities impact user experience.

## Appendix

Multimedia Appendix 1 Study materials, participant demographics, and multilevel model details.

## Abbreviations

EMA: ecological momentary assessment
ICC: intraclass correlation coefficient
mHealth: mobile health
REDCap: Research Electronic Data Capture
SUS: System Usability Scale
uMARS: Mobile Application Rating Scale: User Version
