# Supplementary material for: Using a Smartwatch App to Understand Young Adult Substance Use: Mixed Methods Feasibility Study
Source: JMIR Hum Factors. 2024 Jun 20;11:e50795. doi: 10.2196/50795 (PMC11224702; doi:10.2196/50795)
Supplement: Multimedia Appendix 1 [file humanfactors_v11i1e50795_app1.docx]

#### Table S1. Description of EMA items that participants were asked about through the SPARC app.

| **Variables** | **Assessment Items** | **Relevant Session** |
| --- | --- | --- |
| Consequences associated with Substance Use [38] | Whether the participants: were able to express feelings more easily, felt more energetic, got a buzz, were in a better mood, were more sociable, felt relaxed, did something that embarrassed them, were rude or obnoxious, couldn’t remember what they did/forgetting what they did, hurt or injured themselves by accident, felt nauseated or vomited, became aggressive, had a hangover | Session 1 |
| Stress | Occurrence of stressful events (yes/no), and rating stress levels (0 - 100) of prior day |  |
| Sleep | Time at which participants went to sleep and woke up |  |
| Mood [35] | Rating the following on a scale of 0 - 6: Tired - Awake, Content - Discontent, Agitated - Calm, Full of energy - Without energy, Unwell - Well, Relaxed - Tense, Left out - Like I belonged, Unsafe - Safe | All Sessions |
| Substances Used | Alcohol, Marijuana, Cigarettes/cigar/cigarillo, Vaping (e-cig or juuling), Stimulants, Other, None |  |
| Intensity of Substance Use | No. of drinks consumed (0 - 10+) and no. of hits had (0 - 10+) |  |
| Experiences associated with Substance Use [36,37] | How participants felt after consuming alcohol (buzzed, tipsy/”happy”, drunk, or wasted) and cannabis (relaxed, clam/”chill”, high, stoned) |  |
| Social Context (People) | How many people participants were at each selected place (0 - 10+), and who they were with, from the following options: Family, Friends, Significant Other, Roommate, Coworker, Strangers, Other |  |
| Social Context (Place) | Where participants were at, from the following options: Home, Work, Friend’s house, Bar/club, Restaurant, Outdoors, Other |  |
| Social Context (Experiences) | Of people participants were with, what % used alcohol (0 - 100%), and rating following experiences on a scale of 1 (not at all) to 9 (very much):  how often the people they were with teased or said mean things to them, and how much the participants would like to spend time with the group they were with. |  |

####

####

#### Textbox S1. Semi-structured Interview Script

| • What was your experience using an Apple Watch?   - How difficult was it to complete the surveys on the Apple Watch? - Was the font size appropriate? - Was the button size and placing appropriate? - Did you have any physical difficulties in using the watch? - Is it something you felt you were used too? (using the watch in this way)   • Were there specific questions that were harder than others?   - Were there any formats (radio buttons, tap sliders) that were harder than the others? – How time consuming did it feel to complete each survey?   • Were there any barriers to completing each survey?  • Was it disruptive to your daily life?  • Do you think it would have been easier to complete each survey if you completed it on the iPhone?  Why/why not?  • How useful were the notifications?  • Was the notification frequency appropriate?  • If you were designing this study, what would you change or add?  • If you were using substances while completing surveys, did that impact your responses in anyway?   - Was it harder to understand? - Was it harder to answer?   • Did using the SPARC Watch app increase your awareness of substance cravings?  • Did you use the app in public/social settings? Why/why not?   - Did you feel uncomfortable in any way while doing this?   • Did you have any concerns about your privacy while participating?   - If yes, were there any specific kinds of data that you felt concerned about? - Any specific aspects of app interaction that you felt concerned about? (time, location, social   env?)  • Was the compensation level adequate based on the amount of time you spent completing these  surveys?  • Did you know anyone else who was participating in the study?  • What motivated you to participate in this study? (Probe: new technology, knowing about oneself,  money)  • Do you have any other comments you’d like to make for the research team? |
| --- |

####

####

####

####

#### Table S2. Participant Demographics^^[[1]](#footnote-1)^^.

| **Participant** | **Age** | **College Standing** | **Biological Sex** | **Yearly Family Income** | **Race and Ethnicity** | **Past Month Alcohol Use** | **AUDIT Score** | **iPhone Ownership Duration** | **Apple Watch Ownership Duration** |
| --- | --- | --- | --- | --- | --- | --- | --- | --- | --- |
| P1 | 24 | Graduate Student | Male | $40, 001−60,000 | Asian | 2-4 times a month | 2 | 2-3 years | <1 year |
| P2 | 21 | Undergraduate,  4th year | Female | $100,001 or more | White | 2-4 times a month | 4 | 5+ years | 2-3 years |
| P3 | 20 | Graduate Student | Male | $100,001 or more | Prefer Not to Say | 2-3 times a week | 5 | 5+ years | 3-5 years |
| P4 | 21 | Undergraduate,  4th year | Female | $100,001 or more | White | 2-3 times a week | 6 | 5+ years | 1-2 years |
| P5 | 21 | Undergraduate,  4th year | Female | $100,001 or more | White | 2-4 times a month | 3 | 5+ years | 2-3 years |
| P6 | 21 | Undergraduate,  3rd year | Male | $100,001 or more | White | 2-4 times a month | 4 | 2-3 years | 2-3 years |
| P7 | 25 | Undergraduate,  4th year | Female | $100,001 or more | White | 2-3 times a week | 15 | 5+ years | 2-3 years |
| P8 | 25 | Graduate Student | Female | $20, 001−40,000 | White | 2-3 times a week | 9 | 5+ years | 3-5 years |
| P9 | 20 | Undergraduate,  3rd year | Female | $100,001 or mor | Asian | 2-4 times a month | 6 | 5+ years | 1-2 years |
| P10 | 21 | Undergraduate,  4th year | Female | $100,001 or mor | White,  Hispanic or Latine | 2-3 times a week | 8 | 5+ years | 2-3 years |
| P11 | 24 | Graduate Student | Female | $10, 000−20,000 | Asian | 2-4 times a month | 2 | 5+ years | <1 year |
| P12 | 21 | Undergraduate,  3rd year | Female | $40, 001−60,000 | Asian | Monthly or less | 1 | 1-2 years | 2-3 years |
| P13 | 25 | Graduate Student | Male | $20, 001−40,000 | Asian | Monthly or less | 2 | 5+ years | <1 year |
| P14 | 22 | Undergraduate, 4th year | Female | $80, 001−100,000 | White | 4 or more times a week | 10 | 5+ years | 1-2 years |
| P15 | 22 | Undergraduate, 4th year | Male | I don’t know/Prefer not to Say | Black or African American | Monthly or less | 1 | 5+ years | 1-2 years |

####

#### Table S3. Summary of model predicting likelihood of responding. Sessions were significant predictors of participants’ likelihood to respond. All significant effects are in bold.

|  | | | **95% CI Odds Ratio** | | |
| --- | --- | --- | --- | --- | --- |
| **Predictor** | **B (SE)** | **P** | **Lower** | **Odds Ratio** | **Upper** |
| Session 1 | 1.04 (0.28) | **< 0.01** | 1.65 | 2.84 | 4.91 |
| Session 2 | -1.45 (0.22) | **< 0.01** | 0.15 | 0.24 | 0.36 |
| Session 3 | -1.73 (0.23) | **< 0.01** | 0.11 | 0.18 | 0.28 |
| Session 4 | -1.96 (0.23) | **< 0.01** | 0.09 | 0.14 | 0.22 |
| Session 5 | -3.14 (0.28) | **< 0.01** | 0.03 | 0.04 | 0.07 |

####

#### Table S4. Summary of model predicting likelihood of reporting substance use. Sessions were significant predictors of participants’ likelihood to respond. All significant effects are in bold.

|  | | | **95% CI Odds Ratio** | | |
| --- | --- | --- | --- | --- | --- |
| **Predictor** | **B (SE)** | **P** | **Lower** | **Odds Ratio** | **Upper** |
| Session 1 | -2.23 (0.61) | **< 0.01** | 0.03 | 0.11 | 0.36 |
| Session 2 | -0.45 (0.50) | 0.36 | 0.24 | 0.63 | 1.68 |
| Session 3 | -0.73 (0.52) | 0.16 | 0.17 | 0.48 | 1.34 |
| Session 4 | 0.03 (0.48) | 0.95 | 0.40 | 1.03 | 2.61 |
| Session 5 | 1.70 ( 0.67) | **< 0.01** | 1.47 | 5.49 | 20.42 |

1. This table presents a portion of demographics data collected through the baseline assessment. In our analysis of this data, 67% of participants report that their biological sex is female. Participants ranged from 20 to 25 years of age (Mean = 22.20, SD = 1.86). All participants are college students, and our sample consisted of both undergraduate and graduate students (𝑛_𝑢𝑛𝑑𝑒𝑟𝑔𝑟𝑎𝑑𝑢𝑎𝑡_𝑒 = 10, 66.67%; 𝑛_𝑔𝑟𝑎𝑑𝑢𝑎𝑡𝑒_ = 5, 33.33%). Only one participant reported that they were affiliated with greek organizations (fraternities or sororities) on campus. In terms of race, a majority of participants identified as White (n = 8, 53.33%) or Asian (n = 5, 33.33%), one participant identified as Black or African American, and one participant preferred not to answer a question about their race. Only one of the fifteen participants identified as Hispanic or Latine. Past-month alcohol use among participants was at four levels: 4 or more times a week (n = 1,6.67%), 2-3 times a week (n = 5, 33.33%), 2-4 times a month (n = 6, 40%), and monthly or less (n = 3, 20%). Alcohol amounts consumed on a typical drinking day were distributed across three levels: 1 or 2 drinks (n = 10, 66.67%), 3 or 4 drinks (n = 4, 26.67%), and 7 to 9 drinks (n = 1, 6.67%). Five participants reported that they consume six or more drinks on a single occasion at least monthly. To further assess alcohol consumption and drinking behaviors, we used the Alcohol Use Disorders Identification Test (AUDIT) as a part of the baseline survey. Our analysis indicates that most of our participants consume alcohol in a non-hazardous manner, but our 25% of our sample does include participants that consume alcohol in a harmful manner. 73.33% of participants (n = 11) had scores between 1-7, which suggests low-risk consumption according to WHO guidelines, 3 participants had scores between 8-14, which suggests hazardous or harmful alcohol consumption, and 1 participant had a score of 15, which is the threshold for indicating the likelihood of alcohol dependence. Overall, the fifteen participants included in our analysis present a range of substance use frequencies, intensities, and behaviors. [↑](#footnote-ref-1)
